# Influences of *Ruminococcus bromii* and *Peptostreptococcaceae* on voluntary exercise behavior in a rodent model

**DOI:** 10.3389/frmbi.2024.1389103

**Published:** 2024-05-08

**Authors:** Matthew Rusling, Anisha Karim, Avi Kaye, Chia-Ming Jimmy Lee, Lauren Wegman−Points, Victoria Mathis, Thomas Lampeter, Li-Lian Yuan

**Affiliations:** ^1^Department of Pharmacology and Physiology, Des Moines University, West Des Moines, IA, United States; ^2^College of Osteopathic Medicine, Des Moines University, West Des Moines, IA, United States; ^3^Department of Medicine, The Metrohealth System, Case Western Reserve University, Cleveland, OH, United States

**Keywords:** exercise behavior, microbiome, *Ruminococcus*, *R. bromii*, *Peptostreptococcaceae*, alpha diversity

## Abstract

**Introduction:**

This study investigates the relationship between the gut microbiome and voluntary exercise, focusing on wheel running activity in a rat model. The gut microbiome plays a crucial role in host physiology, homeostasis, and behavior. Alterations in the gut microbiome have been linked to various pathological states and health conditions, including obesity.

**Methods:**

Given the strong association between physical inactivity and obesity development, our study aimed to identify microbiome factors associated with elevated levels of voluntary exercise. Male Sprague Dawley rats were used in the 4-week exercise paradigm in which voluntary wheel running behavior was monitored alongside weekly microbiome sampling from fecal pellets.

**Results:**

We observed individual differences in running activity among the cohort. Significant positive correlations in running distance were identified across the 4-week time course, suggesting that running activity ranking was largely preserved. Furthermore, earlier running activity emerged as a potential predictor for subsequent running behaviors. Analysis of gut microbiome revealed that alpha diversity was positively correlated with daily running distances, with significant differences in beta diversity observed between high and low running groups. Taxonomic analysis showed distinct abundance differences between running and sedentary conditions, particularly in the *Ruminococcaceae* and *Peptostreptococcaceae* families.

**Discussion:**

Our results suggest that the microbiome composition changes significantly early in exercise exposure, potentially influencing exercise behavior. *Ruminococcaceae*, particularly *R. bromii*, was identified as a significant contributor to exercise adaptation, while *Peptostreptococcaceae* was inversely related to running performance as well as alpha diversity. This study underscores the potential of the gut microbiome as a modulator of exercise behavior. Future research should focus on the biological mechanisms linking microbiome changes to exercise adaptation, with *R. bromii* and *Peptostreptococcus* as promising candidates for influencing exercise behaviors through future interventional studies.

## 1 Introduction

The human gut microbiome (GMB) – composed of roughly 150 times the gene quantity of the human genome alone – is integrally involved in host behavior, physiologic homeostasis, and sympathetic nervous system activity through a reciprocal gut-brain axis (Dinan and Cryan, 2017). Consequently, alterations in the GMB can inhibit or promote numerous pathological states such as diabetes, irritable bowel disease, depression, and various autoimmune conditions (Kelly and Mulder, 2012). Unsurprisingly, specific GMB conditions are also associated with obesity, demonstrating consistent compositional changes and decreases in alpha diversity based on diet (Ribeiro et al., 2022). This observation is further corroborated by the reduction in microbes involved in regulating fat mobilization, carbohydrate metabolism, insulin responsiveness and immune modulation with high-fat diets and increased adiposity (Asnicar et al., 2021). For example, the GMB has been proposed to influence adiposity through the endocannabinoid (eCB) system (Muccioli et al., 2010) and other mechanisms.

As obesity is of global interest in health populations, we should consider its contributing factors. Limited physical activity has consistently been shown to be a central contributor to the obesity epidemic; conversely, increasing physical activity is crucial to improving overall health (Swift et al., 2014). Other population level health benefits of exercise find that moderate to high physical activity alone is 40% more protective against cardiovascular events than other risk factors such as blood lipids, hypertension and diabetes (Joyner and Green, 2009). Given the broad benefit of exercise on health, even factors with small effect sizes influencing this behavior could have outsized population level benefit. There is increasing evidence that a portion of exercise’s protective effects are mediated by alterations in the GMB (Joyner and Green, 2009; Monda et al., 2017; Taniguchi et al., 2018)}. Consistently, exercise has been shown to enhance beneficial conditions such as increased alpha diversity in obese rodent models (Denou et al., 2016; Yang et al., 2021), as well as in humans (Ortiz-Alvarez et al., 2020).

Multiple mechanisms have been proposed for GMB based improvements in exercise capacity, such as exercise associated increases in microbes producing anti-inflammatory producing bioactives such as butyric acid (Yu et al., 2019). Similarly, there are correlations between exercise performance and metabolomic propionic acid production along with other short chain fatty acids (SCFAs) (Muccioli et al., 2010; Sales and Reimer, 2023). Other studies illustrate connections between physical activity and increased microbiota metabolomic representation of tricyclic acid cycle genes (Rojas-Valverde et al., 2023), glycolysis and oxidative phosphorylation which directly enhance host metabolic performance and capacity (Hintikka et al., 2023). Lastly, analgesic effects of exercise, effecting tolerance, are also shown to be potentially dependent on microbial communities, such as those that stimulate peripheral cannabinoid receptor 1 (CB1)-mediated signaling (Koltyn et al., 2014). As an example, increased CB1 receptor modulation has previously been identified as a potential motivational factor in voluntary physical activity in mammal models including humans and that microbiome knockout has a similar effect as pharmacologic receptor blockade on exercise behaviors (Raichlen et al., 2012; Dohnalova et al., 2022).

To date, it has been well established that exercise induces changes in the microbiome in both human and non-primate models. The next question in studying this domain is how well microbiome variance associates with voluntary exercise behaviors. In this investigation we sought to describe how microbiome variance was associated with running activity in a voluntary exercise animal model. This was accomplished by correlating the composition of gut microbiota (GMB) with discrete running distances. Additionally, we subdivided the experimental conditions into high and low running based on their running performance. This categorization distinctly differentiated between two groups: high-distance runners and low-distance runners.

## 2 Methods

### 2.1 Animals

7-week-old male Sprague Dawley rats were obtained from Charles River Laboratories (Wilmington, MA) and were housed in temperature- (22°C) and light- (12/12 h dark/light) controlled animal quarters. The rats had free access to standard laboratory rat chow and drinking water. Experimental procedures were conducted in strict adherence to the National Institutes of Health Guide for the Care and Use of Laboratory Animals of the National Research Council of the (U.S.) National Academies and were approved by Des Moines University Institutional Animal Care and Use Committee (IACUC). A summary of the experimental design is found in Results 3.1.

#### 2.1.1 Voluntary wheel running

After a 7-day acclimation period, rats at the age of 8-week were randomized into the sedentary control (n=3) and wheel running group (n=8). Sedentary rats were individually housed in standard rat cages. For the running group, rats were housed individually in polycarbonate living chambers (40.64 x 50.80 x 20.96 cm) equipped with stainless steel lids and running wheels with a circumference of 1.10 meters (Scurry Rat Activity Wheel with Living Chamber, Lafayette Instrument, Lafayette, IN). Scurry Rat Activity Counters were mounted to the wheels and connected to the Scurry Interface for Animal Activity. The interface was connected to a computer, and the use of each running wheel was reported in real time and stored in the Scurry Activity Monitoring Software (Lafayette Instrument, Lafayette IN). Wheel revolutions were recorded continuously throughout the experiment and translated to running distance based on the wheel size. The duration of running (or time spent on running) is determined as the total number of minutes with at least 1 wheel rotation. Speed is the average distance per minute across all minutes with at least 1 wheel revolution.

### 2.2 Genomic DNA isolation & sequencing

For both sedentary and running rats, fecal samples were collected aseptically at week 0 (before running rats were given access to wheels) and then weekly for the next 4 weeks. Collected fecal pellets were placed in individually labeled sterile tubes, then stored at -80C until sample processing. Genomic DNA was isolated using PowerSoil-htp 96 Well Soil DNA Isolation Kit (MoBio, Carlsbad, CA). The protocol for DNA isolation provided by the manufacture was followed with the exception that the initial vortex step was extended to 20 minutes to thoroughly homogenize the samples. PCR amplification of the V4 variable region of the 16S rRNA gene using V4 region specific primers (515F-806R) and amplicon sequencing were performed by the Institute for Genomics & Systems Biology at the Argonne National Laboratory (Argonne, IL) using the Illumina MiSeq platform.

### 2.3 Statistical approach

#### 2.3.1 VWR analysis

Daily running behaviors between models reached an asymptotic point within the final week of the study. The average daily distance run during the final 7-day period was used to quantify terminal running behaviors. This identified 3 animals that appeared to have significantly higher terminal running behavior than the other models. Terminal behaviors of these high performing models were tested with ANOVA to confirm that this subjective difference demonstrated a significant separation of models into ‘high’ and ‘low’ running groups. Given that this was an exploratory study, we applied both group comparisons using this high and low runner assignment, as well as comparison between bacterial abundance and continuous VWR distances to identify GMB candidates associated with exercise behavior, as described below.

#### 2.3.2 Pre-processing

Data was processed using QIIME2-2022.11. Reads were de-noised and paired using DADA2, and further downstream analysis was performed in R. Demultiplexed samples produced 100,965 ± 51,474 reads that passed filtering checks. Reads were trimmed at base 5 for both forward and reverse reads and truncated at 238 and 230 bases respectively. Denoising and merging yielded 76,910 ± 39,049 reads per sample. Three samples were found to have below threshold read counts and were discarded. After sample removal the dataset included in analysis contained 81,194 ± 35,647 reads per sample that passed denoising, merging and chimera checks. Singleton features and those observed in only 1 sample were removed, yielding 81,180 ± 35,655 features per sample. Downstream feature composition processing was performed on a dataset where microbiome features present in fewer than half of samples were removed to avoid false positive results from outlier low abundance/low frequency taxa.

#### 2.3.3 Alpha & beta diversity

Alpha diversity metrics were produced within the QIIME2 environment for Shannon, Simpson and Chao1 metrics. Shannon diversity was evaluated for its longitudinal change and relationship with daily distance using fixed slope random intercept models. Beta diversity was analyzed using QIIME2 ADONIS.

#### 2.3.4 Generalized Least Squares modeling

Analysis was performed using the *gls()* function included in the R *nlme* package evaluating the expression: *GLS([abundance]_baseline_ ~ final distance run)* (Pinheiro Jc and R Core Team, 2023). Generalized Least Squares (GLS) was selected due to features being non-normally distributed yielding heteroscedastic models with repeated measures data containing high degrees of within-individual correlation at every taxonomic level. GLS models used in this study used the correlation structure *Compound symmetry correlation (form = 1/ratid)*, where *ratid* was the unique label used for each animal. These models were also chosen as other methods, such as T-test or ANOVA, (while able to detect group effects and interactions), are less powerful in accounting for within individual autocorrelation and do not detect the direction of an interaction; especially in our dataset which involves the testing of multiple continuous variables.

#### 2.3.5 Baseline analysis

Baseline analysis used GLS model to compare microbiome abundance at the start of the experiment (time 0) with VWR distances averaged from the final week of the experiment. This was iterated across all observed taxa from the phyla to genus level to identify what baseline feature variance was associated with terminal behavior.

#### 2.3.6 Compositional comparison between running and sedentary conditions as well as high and low runners

To differentiate features which had a temporal effect across both running models and sedentary controls, we evaluated GLS([abundance] ~ condition_(sedentary/running)_ *time) accounting for within individual autocorrelation in the model and treating time as ordinal factors. Acknowledging that this decision has limitations, this was done to identify which features were significantly different and at which specific timepoint these changes occurred. If time were to be treated as a continuous variable, we would not have been able to identify at what timepoint condition or temporal effects occurred. Features which were significant for their time*condition interaction, but not for *time* alone are identified as meaningful. Features meeting this criterion are identified as relevant, as this criterion demonstrates that the effect is not attributable to a confounder affecting both conditions. This same approach was taken to compare GLS([abundance] ~ run group_(low/high)_*time).

#### 2.3.7 Compositional variance associated with daily running distances

To identify how variance within the GMB affected observed variance in running behavior, GLS*([abundance] ~ daily running distance)* on the day of fecal pellet collection was evaluated. Given that multiple comparisons were being made across taxa with different magnitudes of abundance, predictor variables were centered and scaled with Z-score using *scale([abundance])* in the base R package, which were calculated within each taxon (R Core Team, 2022). This was done so effect sizes between features with different baseline abundances could be compared. Results were then filtered to exclude models which did not reach the threshold for significance (p<0.05). Significant models were reported. For higher taxa which were able to be classified at lower levels, the lowest level of classification reaching significance was described in the results section but included in the figures.

## 3 Results

### 3.1 Voluntary wheel running behavior

Voluntary wheel running (VWR) is a natural and rewarding behavior to rodents (Greenwood et al., 2011; Meijer and Robbers, 2014), serving as a model of voluntary exercise that primarily occurs during the dark phase (Yamanaka et al., 2013). Previous studies reveal that over a 6-week exposure to running wheels, running distance initially increases for around 3 weeks (acquisition phase) and then stabilizes for the next 3 weeks (maintenance phase) (Greenwood and Fleshner, 2019).

**Figure 1:**
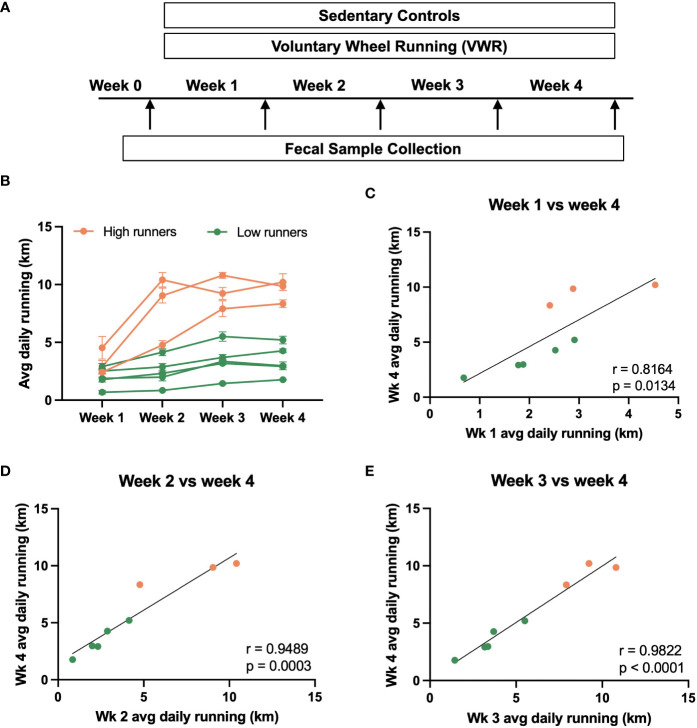
Voluntary running behavior of male rats. **(A)** Summary of experimental design. Rats were subjected to a 4-week voluntary wheel running (VWR) regimen, during which the daily running distance of each rat was monitored. Sedentary rats without access to the running wheels served as controls. Fecal pellets were collected from individual rats at the end of each week, with Week 0 representing the timepoint just before the initiation of VWR exposure. **(B)** Average daily running distance of individual rats over the experimental duration (week 1, 2, 3, and 4). Each line represents an individual rat. Based on the final week running performance, the VWR group was further divided into high vs. low running groups. **(C–E)** Correlational analysis depicting the relationship between the average daily running distance of individual rats in week 4 against that of week 1, week 2, and week 3. Trendlines indicate statistically significant relationships, with R- and p-values specified on each plot.

In our study, we implemented a 4-week voluntary wheel running (VWR) regimen (**Figure 1A**), monitoring the daily running distance of each rat. Sedentary rats without access to running wheels served as controls. Fecal pellets were collected weekly, with Week 0 representing the period just before initiating VWR exposure. As seen in other studies (Yamanaka et al., 2013), there were individual differences in running activity within the cohort.

Male rats provided free access to running wheels exhibited low initial running activity, but after four weeks, running distance varied substantially between cohort members (**Figure 1B**). Using a 6 km/day threshold based on final week running performance, we categorized the VWR group into high and low running sub-groups for future microbiome analysis. Positive correlations in running distance were consistently observed over the 4-week time course (**Figures 1C–E**). Notably, the average daily running distance of the final week (week 4) strongly correlated with that of weeks 2, 3, or 4 (n=8, p<0.05). These findings collectively suggest that running activity ranking remains relatively consistent over time, with earlier running activity emerging as a potential predictor for subsequent running behaviors.

### 3.2 Alpha diversity correlates with daily running distance

Kruskal Wallis pairwise comparisons were performed between sedentary, high, and low running groups. Pooling all timepoints, there was no significant differences between the alpha diversity of these groups. Temporally, there was a significant increase across running & sedentary conditions at timepoints 3 and 4. Shannon diversity was correlated with daily distances run on the day of sample collection (GLS, Int. -204±1800, Est. 606±299, RSE =3197, p<0.05), however baseline alpha diversity was not correlated with final running distances.

### 3.3 Beta diversity reveals significant differences between high and low runners

Pairwise permanova was used to compare pooled timepoints using Weighted UniFrac which found significant differences between high and low run groups (F = 3.8, p-value < 0.001) as well as the high run group and sedentary group (F = 4.7, p-value < 0.01). Low running & sedentary groups were not significantly different from each other.

### 3.4 Taxonomic abundance differences between running and sedentary models

**Figure 2:**
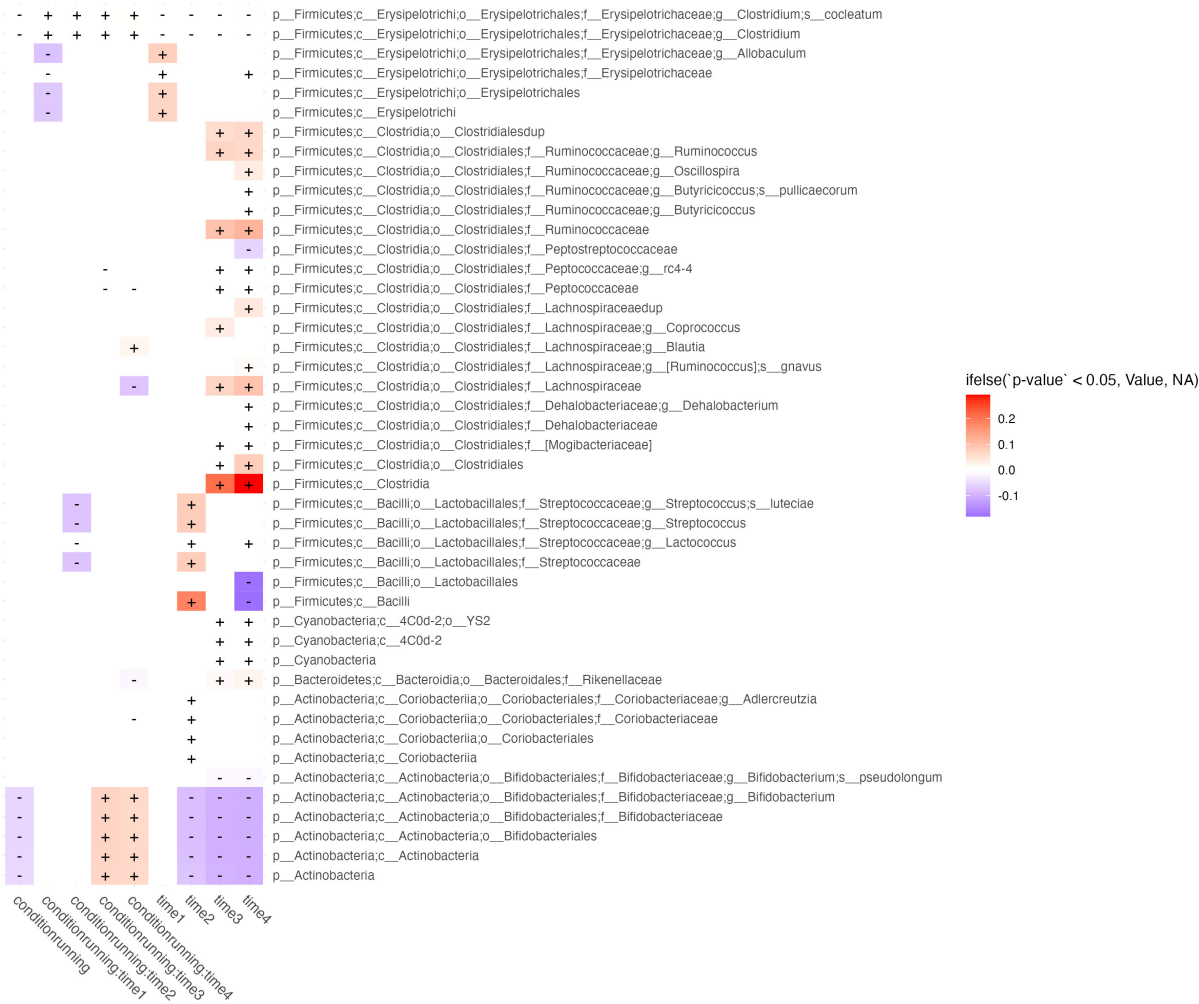
Longitudinal effects of exercise on microbiome abundance. Taxa (rows) which are significantly affected by at least 2 periods of time, running exposure vs. sedentary models, or their interaction (columns) are shown in this figure. Cells correspond to features effected by running exposure (controlled against the sedentary group) across time (columns labeled “*running*”), running exposure at discrete timepoints (columns showing predictor interactions labeled *run group:time_n_*), and temporal effects irrespective of running exposure (columns labeled *time_n_*). Cells are shaded by whether the effect of the predictor on feature abundance is in the negative (blue) or positive (red) direction, and fill intensity reflects the magnitude of the effect size.

GLS was used to evaluate the effects of [Abundance] ~ condition_running/sedentary_ * time_1,2,3,4_ (**Figure 2**). We were interested in features with significant interactions between predictors and without significant time effects at the same period. Features with only a condition*time interaction are those in which a true running effect is observed, instead of a temporal confounder common between conditions. This method showed that across timepoints the running condition had lower levels of *B. Bifidobacteria*, however, at weeks 3 & 4 the abundance of this taxa increased relative to its change within the sedentary models.

**Figure 3:**
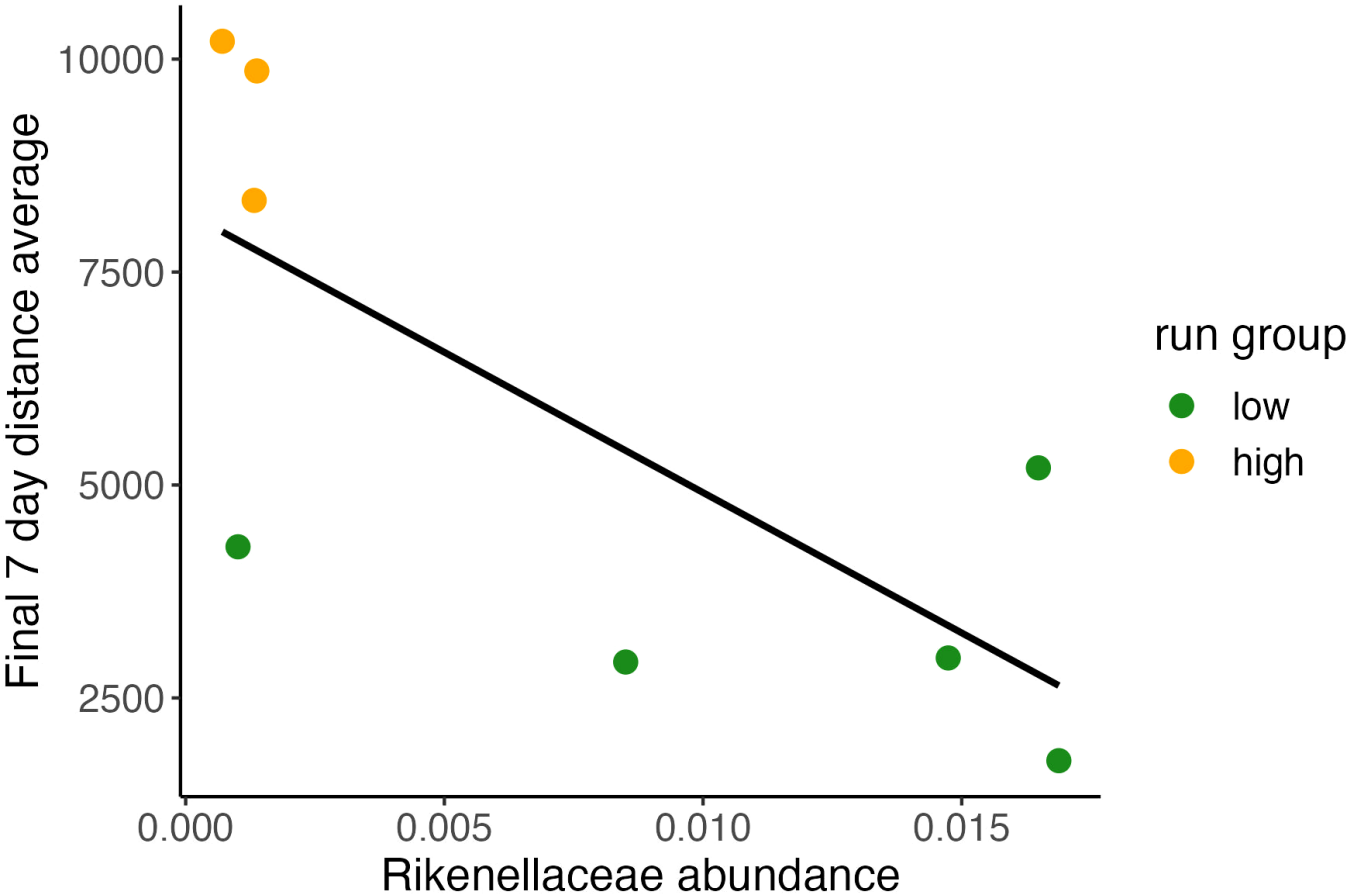
Baseline relative abundance of Rikenellaceae predicting final running distance. Relationship between individual final 7-day running average plotted and relative abundance value of Rikenellaceae shows significant correlation between baseline abundance and terminal running distances. (Pearson Correlation, (*y* = 8.21*10^3^ *x* – 3.3*10^5^), R2 = 0.54, P<0.05).

Various subclassifications of the *Streptococcacea* family were also significantly decreased at week 2 in the running condition. Various subclassifications of *Erysipelotricaceae* were decreased in week 1 of the running condition. Temporal effects, irrespective of running, were observed mostly in growth of *Firmicutes*, represented mostly features of the *Clostridiales* order over the course of the study (**Figure 3**).

### 3.5 Baseline biome abundance prediction of terminal running distance

**Figure 4:**
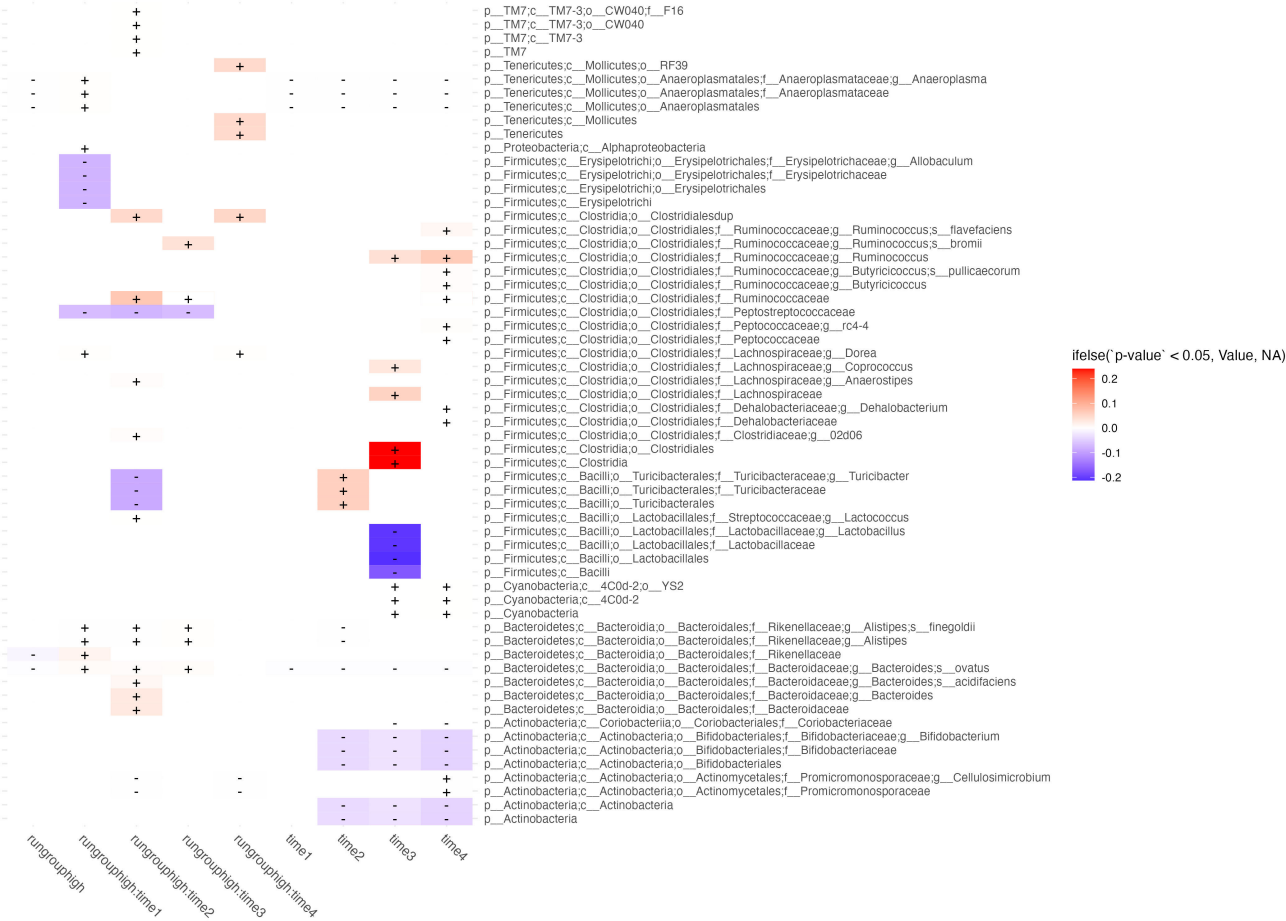
Differential abundance between high and low runners. Taxa (rows) abundance which are affected by either time, runner type (high or low), or their interaction (columns) are shown in this figure. Columns correspond to differential abundance between run groups irrespective of time (column 1, labeled “*run group high*”), group effects at each week (columns 2-5, showing predictor interactions labeled *run group:time_n_*), and temporal effects irrespective of runner type (rightmost 4 columns labeled *time_n_*). Cells are shaded by whether the effect of the predictor on feature abundance is in the negative (blue) or positive (red) direction, and fill intensity reflects the magnitude of the effect size.

For each microbiome feature observed in our dataset we performed a series of models evaluating *[terminal week distance] ~ [abundance]* to identify if baseline abundance predicted future running behavior (**Figure 4**). Only one taxonomic feature, *B. Rikenellaceae*, was found to be nearly significant (Pearson Correlation, R2 = 0.54, P <0.05; final 7-day average distance run = 8.21*[Rikenellaceae_baseline_] + 10^3^). Baseline microbiome condition, possibly apart from *B. Rikenellaceae* abundance, does not predict terminal running behavior. In further analyses we explored whether adaptive changes in the GMB better explain running behavior.

### 3.6 Microbiome comparison between high and low runners reveals differential abundance early in exercise exposure

We ran a series of models evaluating [abundance] ~ run group _low/high_ * time_1,2,3,4_ and present features which were significantly associated with those predictors (**Figure 4**). The most important results are those with significant interactions between high runners and time which were not shared with the time predictor. Features with significant interactions which were decreased in the high running group included: *E. Allobaculum* at week 1, *C. Peptostreptococcaceae* at weeks 1-3, *T. turicibacter* at week 2, and *P. cellulosimcrobium* at weeks 2 & 4. Interactions which showed increased abundance in the high running group included: *R. flavefaciens* at weeks 2 & 4, *R. bromii* at week 3, *C. Ruminococcaceae* at weeks 2 & 3, *B. ovatus* at weeks 1-3. All microbiome features which were different between run groups were consistent in the direction of their effect (positive or negative direction) across all timepoints.

### 3.7 Microbiome variance associated with daily running distances

**Figure 5:**
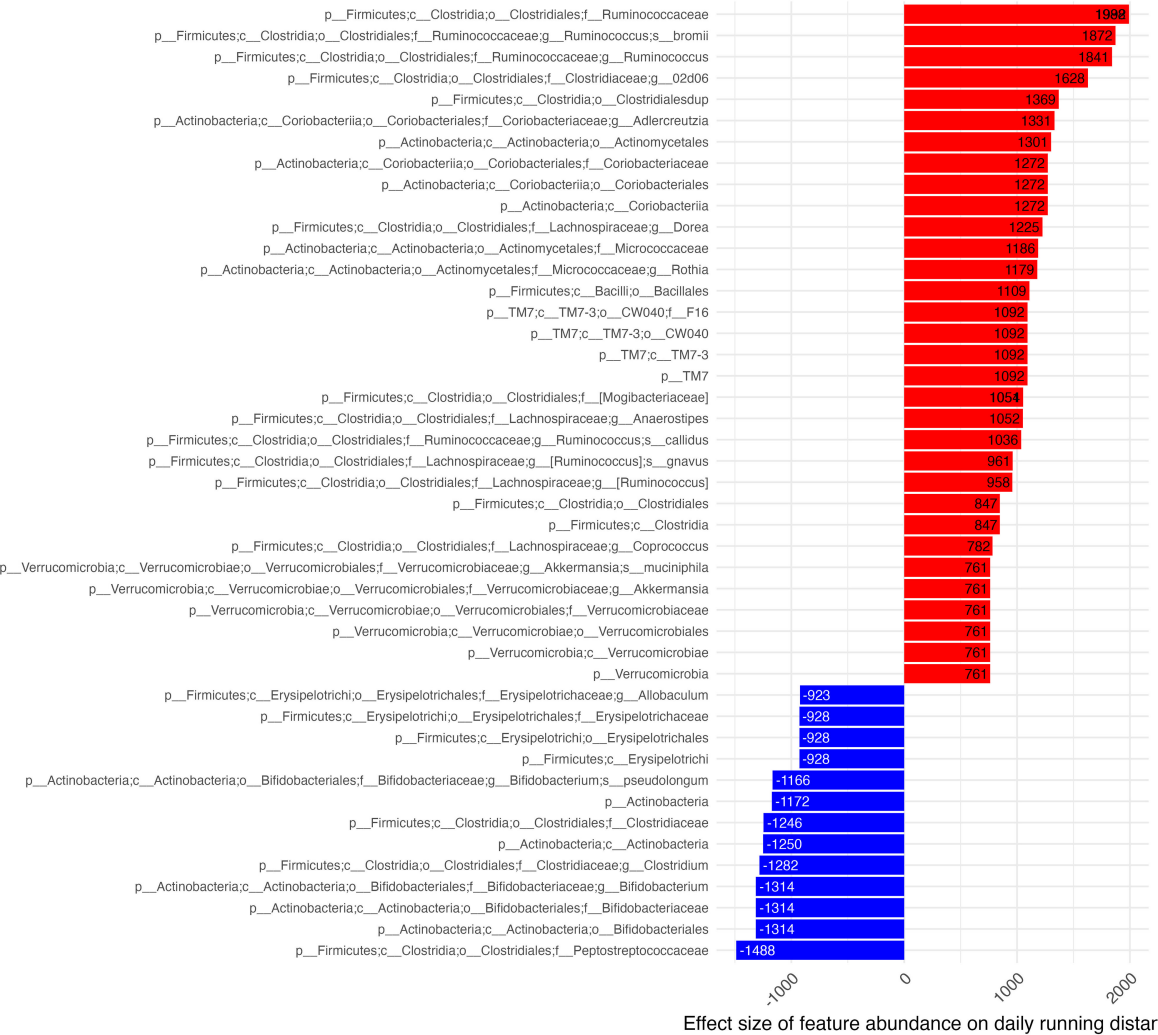
Effect of microbiome features on daily distance. Effect estimates of centered & scaled taxa abundance on daily running distance (x-axis) shown in descending order where red bars identify features associated with increased daily distances and blue bars identify features associated with decreased distance run. Estimate effect size is shown in column tips.

Numerous taxa were identified by GLS models as significantly associated with daily running distances. Of those identified, we report those which were observed to be significant at the deepest level of taxonomic assignment for the significant feature (**Figure 5**). Features with the largest positive effect on daily distances largely belonged to the *Ruminococcus* genus. Features associated with increased distance run were *R. bromii, gnavus* and *collides* and various other subclassifications from the orders: *Clostridiales (Ruminococcaceae, Clostridiaceae (subclassified as 02d06), Lachnospiraceae, Mogibacteriacea), Coriobacteriales (Coriobacteriaceae, Coriobacteriales)*, *Actinomycetales (Micrococcaceae)*, and *Verrucomicrobiales (Verrucomicrobiaceae).*

The feature with the greatest inverse association with daily distance run was *C. Peptostreptococcaceae*. Other features included families of the orders: *Erysipelotrichales (Erysipelotrichaceae), Bifidobacteriales (Bifidobacteraceae)*, and *Clostridiales [Clostridiaceae* (subclassified as *clostridium)].* Features which were able to be more deeply classified from the family level associated *Erysipelotricheae* to its *Allobaculum* genus. *C. clostridium* and *Actinobacteria* were subclassified to the *Bifidobacterium* genus. All features found to be associated with daily distance originated from the *Firmicutes, Actinobacteria* & *Verrucomicrobia* phyla.

### 3.8 Feature selection of microbiome predictors of daily running distance

Having identified family level features associated with daily running distances, these features were included in stepwise feature selection model using exhaustive predictor selection. The initial model, as shown in **Figure 1B**, found that only Ruminococcaceae (Est. 2110±531, p<0.001) and Peptostreptococcaceae (Est. 694±294, p<0.04) were significant predictors. Feature selection produced a final model including 4 predictors: *Ruminococcaceae* (Est. 2414±484, p<0.001)*, Peptococcaceae* (Est. -1258±379, p<0.001)*, Clostridicaeae* (Est. 381±438, p>0.05), and *Micrococcaceae* (Est. 740±387, p<0.1). The final model was significant but not superior to the initial model (ANOVA, L. Ratio 2.4, p=0.9) despite 80% reduction in predictors. Final model Rho=0.69, residuals were evenly distributed, and ran as a linear model with these same predictors had an adjusted R2 value was 0.494. For this dataset, daily running distances were best explained by the following 5-predictor model:


Daily distance=2414*Ruminococcacea+381*Clostridicaeae+740*Micrococcaceae+381*Clostridicaeae−1258*Peptococcaceae+3049

### 3.9 *Ruminococcaceae* and *Peptostreptococcaceae* as behavioral predictors

Given that our analysis to this point has identified *Ruminococcaceae* and *Peptostreptococcaceae* as prominent contributors to both run group and daily running distance, we focused our next set of analyses on these two families. Comparing *Ruminococcocus* abundance over time between sedentary and running groups we found that there was a significant temporal effect, but that running exposure did not significantly affect abundance. Within the running models, by evaluating *Ruminococcus* ~ time*running group, we identified a significant time effect and significant increase in abundance among the high running group at week 2.

**Figure 6:**
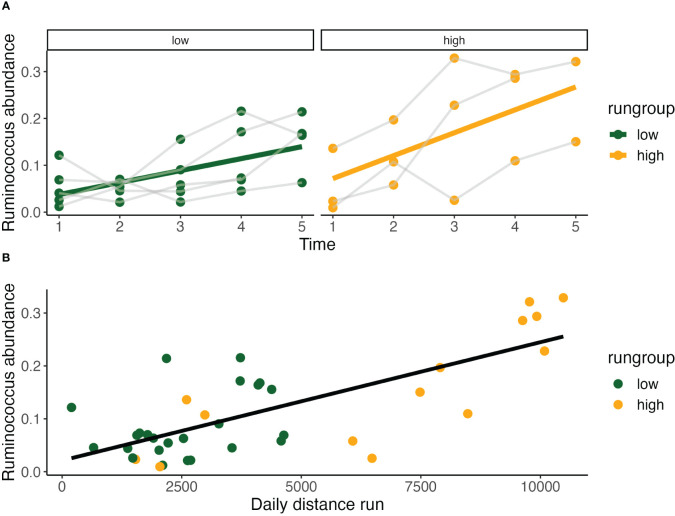
Relationship of *Ruminococcus* with outcome measures. **(A)** Relative *Ruminococcus* abundance increases significantly in both high (Pearson Correlation: *y* = 0.023 + 0.049*x*, R2 = 0.36, P<0.05) and low run groups over time (Pearson Correlation: *y* = 0.012 + 0.026*x*, R2 = 0.34, P<0.01). Relative abundance is also strongly correlated with daily distance run (Pearson Correlation: *y* = 0.021 + 2.24*10^-5^, R2 = 0.54, P<0.001) **(B)**.

**Figure 7:**
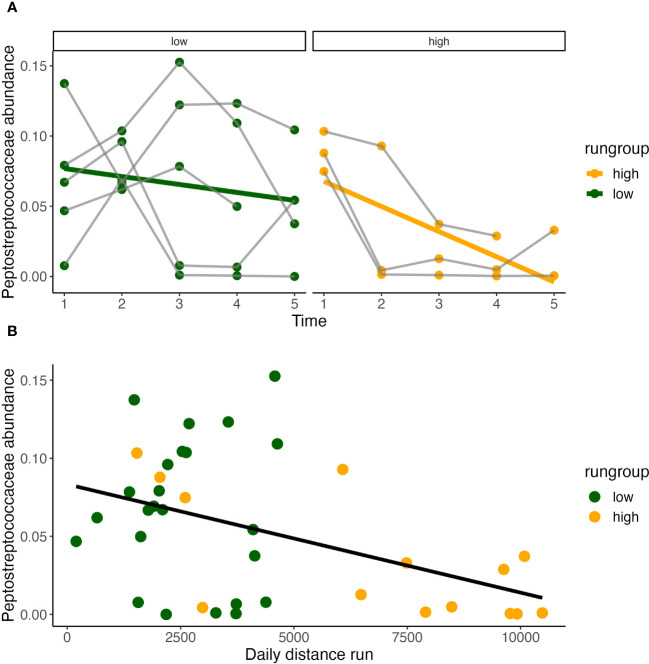
Daily distance correlates with *Peptostreptococcaceae. Peptostreptococecae* abundance significantly decreases over time only in high runners (Pearson correlation, *y* = 0.086 - 0.18*x*, R2 = 0.4, P<0.05) **(A)**, and is inversely correlated with daily distance run **(B)**.

Across both running groups, *Ruminococcus* abundance significantly increases incrementally from baseline at timepoint 2-4, with the greatest difference observed between timepoints 2 (Est. 0.05±0.02, p<0.02) and 3 (Est. 0.10±0.02, p<0.001) representing a doubling in abundance (**Figure 6A**). Notably, it is the change in running distance between timepoints 2-3 that was shown earlier to be the most predictive of terminal running behavior. *Ruminococcus* abundance at various subclassifications was also greater in the high running group across timepoints 2-4 (**Figure 4**). Describing the temporal relationship between running behavior and time, the increase in *Ruminococcus* is not dependent on running behavior alone and increased more rapidly in the high running group (**Figure 7A**) and presents itself as a candidate for increasing spontaneous exercise behavior during exercise adaptation.

Evaluating this relationship between *daily distance ~ Ruminococcus*, we found a highly significant relationship (GLS, Int. 1672±707, Est. 21771±3645, RSE 2139, p < 0.001) with high within-individual correlation (Rho=0.46). Recognizing the limitations of testing repeated measures with simple Pearson Correlation, we found this correlation was highly significant by this measure as well (Pearson Correlation, R2 = 0.54, p < 0.001) (**Figure 6B**).

Temporally, Peptostreptococcaceae ~ time*condition found that abundance decreased over time in both running and sedentary models. Within the running condition, a temporal effect was only observed within the high runners (**Figure 5**) and decreased significantly over time (**Figure 7A**). *Peptostreptococcaceae* showed a highly significant inverse relationship with daily distance (GLS, Int. 4605±902, Est. -26722±7506, RSE 298*7*, p<0.001) (**Figure 7B**).

## 4 Discussion

### 4.1 Evidence of microbiome composition influencing voluntary exercise behavior

Over the 4-week study, we observed variable running behaviors between individuals. Group separation into high and low running groups occurred within the first 2 weeks of adaptation and running distance changes between the first 2 weeks (rather than later differences) best predicted terminal running behavior. This finding implies that the early phase of exercise adaptation, especially the first 2 weeks, is the most important period of GMB adaptation predicting the degree of terminal behavior given unstructured, unprompted exercise. Other work has shown that most adaptation occurs within the first 3 weeks of exposure; adaptation occurs quickly after starting exercise before reaching a plateau (Murias et al., 2010). Our study showed that GMB changed most drastically early in exercise exposure coinciding with this period of rapid physiologic adaptation. Future studies may consider interventional designs to test whether fecal transplant of high and low runners influences running behaviors in the receiving animal models.

During exercise, intraluminal niche conditions change rapidly with increases in IgA production and pH due to anaerobic metabolism of the endothelium and adjacent tissues, while bile acid release, blood flow, and oxygen availability decrease (Wegierska et al., 2022). The downstream effect is an alteration in microbiome composition and metabolism. The extent of how these stressors change the microbiome are dependent on pre-existing composition and function, with some population structures being more resilient than others. Gut permeability is an example of this and is elevated when microbiome states exist that are either 1) more susceptible to disruption caused by changes in the environment, or 2) more inflammatory and harmful to the host at baseline. Consistently, research has shown that exercise causes such increases in permeability, even immediately after exercise (Chantler et al., 2021).

Exploring regulation of gut permeability by GMB composition, we propose that exercise-induced increase in AMPK signaling may be a possible candidate for future research (Macinnis and Gibala, 2017) as AMPK signaling has been found to increase on a similar timeframe as exercised induced increases in gut permeability (Gibala et al., 2009). The connection between these systems may be through lipopolysaccharide (LPS, a biomarker of gut permeability) activation of skeletal muscle TLR4 receptors, resulting in downstream AMPK activation (Fujiyoshi et al., 2022). Preceding biologic events such as AMPK that trigger the cascade of exercise adaptation have so far been under-explored. The multi-directional relationship of host physiology and microbiome structure poses an opportunity to identify novel contributing factors of exercise behaviors.

### 4.2 Biologic relevance of *Ruminococcaceae*

*Ruminococcaceae* is a significant contributor to the abundance of the human microbiome (and in other animal models) and is commonly found in the mucosa, where it plays a key role in forming a ‘mucosal biofilm’ barrier that shields the host endothelium from luminal activity (De Weirdt and Van De Wiele, 2015). *Ruminococcaceae* actively produces butyrate (Gu et al., 2022), neuropeptides including angiotensinogen, leptin, fibroblast growth factor (FGF), glucagon-like peptide-1 (GLP1) in the metaproteome, and metabolic regulators such as adiponectin (Blanco-Miguez et al., 2019; Galie et al., 2021).

Two *Ruminococcaceae* subclassifications are highly represented in the microbiome: *R. bromii* and *R. gnavus*. The metabolic functions of these two species are very distinct. Adaptive behaviors associated with *Ruminococcaceae* in this study were almost entirely attributable to *R. bromii* abundance.

*R. bromii* is one of the dominant strains in the microbiome and serves the important function of degrading particularly resistant starches (RS) using highly specialized and unique amylases that liberate glucose for host metabolism (Ze et al., 2015). In RS rich co-culture with *R. gnavus*, *R. bromii* is also found to be a strong Tryptophan (Trp) synthesizer (Crost et al., 2018).The *R. bromii* is the *Ruminococcaceae* subclassification which produces large amounts of butyrate, and unlike its neighbor *R. gnavus*, may be protective against atopic disorders (Sasaki et al., 2022).

Biologically, *R. bromii* presents three promising mechanisms of interest regarding the relationship between exercise behavior and its abundance: 1) increased Trp bioavailability due to increased *de-novo* synthesis, 2) increased butyrate and acetate SCFA, and 3) increased energy bioavailability through greater liberation of glucose from resistant starches. Further work evaluating the effects of *R. bromii* may consider whether *R. bromii* abundances may impact Trp levels in host peripheral and CNS tissue. *R. bromii*, while a difficult commensal microbe to produce at scale may be a target of future commercial interest. Given the mechanisms described above of *Ruminococcaceae* metabolism, we found compelling evidence that *Ruminococcaceae* is a candidate for the meaningful mechanistic relationship between the microbiome and exercise.

On the other hand, *R. gnavus* serves as an irritant mucin degrader (Crost et al., 2018) and a proposed superantigen, activating the adaptive immune system and found to be up regulated in allergic and autoimmune disorders (Bunker et al., 2019). In the large cohort HUNT study, body fat and C-reactive protein (CRP) were significantly greater in individuals with higher *R. gnavus* abundances (Grahnemo et al., 2022). Across studies spanning inflammatory pathologies, *R. gnavus* is identified as a strong candidate biomarker for having a maladapted microbiome state. In this study, we hypothesize that the increase in *R. gnavus* in the high running group is due to its opportunistic relationship with *R. bromii*.

### 4.3 *Peptostreptococcaceae* as a potential modulator of exercise behavior

*Peptostreptococcaceae* is located within the colonic mucosa and is a lactic acid producing bacteria (De Weirdt and Van De Wiele, 2015). In probiotic studies, *Peptostreptococcaceae* abundance has been inversely correlated with the establishment of the supplemented species, as well as the catabolism of amino acids (Zaccaria et al., 2023). *Peptostreptococcaeae* may decrease alpha diversity and protect its niche from competition by shifting local metabolism from glucose to amino acid catabolism, in particular committing Trp to the Indole pathway, reducing host Trp bioavailability (Wlodarska et al., 2017; Zaccaria et al., 2023). *Peptostreptococcaceae* has also been positively correlated with weight gain in obese individuals (Acharya et al., 2023). We hypothesize that such competition against exercise-related microbiome adaptation may have reduced biologic exercise tolerance. While the evidence for *Peptostreptococcaceae* being an adaptive or maladaptive component of the microbiome is mixed, our results suggest that this family may have a negative effect on exercise behavior.

**Figure 8:**
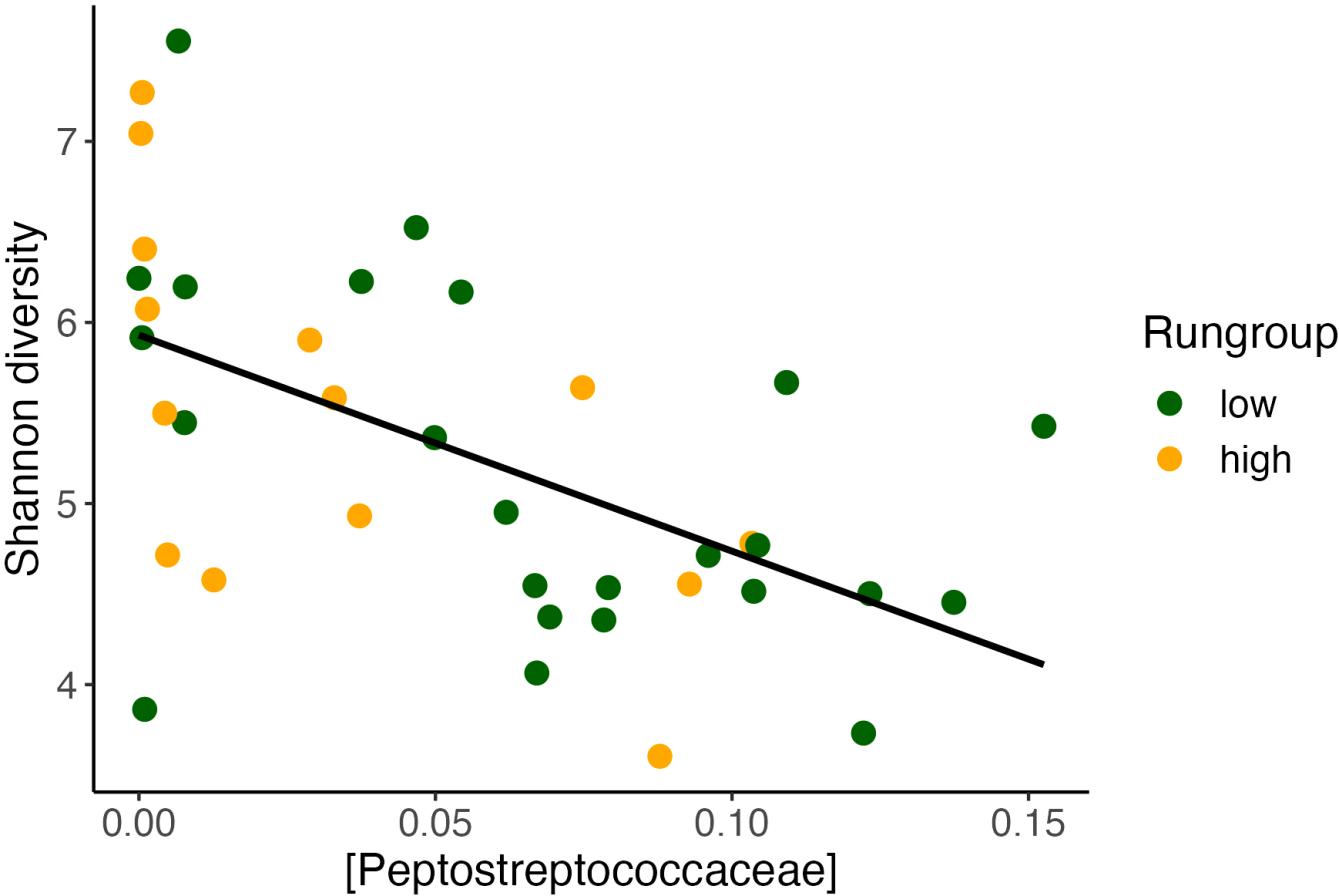
Alpha diversity inversely correlates with *Peptostreptococcaceae.* Alpha diversity found to inversely correlate with Shannon diversity (Pearson’s correlation, *y* = 6.08 – 12.7*x*, R2 = 0.3, P <0.001).

Notably, our alpha diversity results have important consistencies with previous literature. We found that daily running distance was correlated with higher alpha diversity, which is consistent with previous findings that exercise exposure increases alpha diversity (Yang et al., 2021). *Peptostreptococcus* has been shown to be negatively correlated with alpha diversity in other studies (Zaccaria et al., 2023). Therefore, we evaluated this concept in our current study, also finding a negative correlation between *Peptostreptococcus* and alpha diversity. In this dataset, *Peptostreptococcaceae* was also inversely correlated with Shannon alpha diversity (**Figure 8**), suggesting a reproducible relationship worth further investigation. When comparing the impact on daily distance between Alpha diversity and *Peptostreptococcus*, we observe that the variance in *Peptostreptococcus*, rather than alpha diversity, has a more pronounced effect on daily distance. Additionally, *Peptostreptococcus* exhibits a more significant model and a relatively narrower confidence interval estimate. In conditions where increasing alpha diversity may improve outcomes, suppression of *Peptostreptococcaceae* may prove to be a valid target. While we cannot rule out co-linearity as the cause of this observation, it is possible that *Peptostreptoccocae* impairs the effect of exercise in part by suppressing alpha diversity. Our modeling results suggest that *Peptostreptococcaceae* abundance itself may have a direct effect on exercise behavior. Future GMB research which observes a significant change in alpha diversity in their outcomes could explore whether this *Peptostreptococcaceae* relationship is further conserved.

### 4.4 Limitations of this study

Due to the specific setup of the running wheel cages, we were unable to accurately measure and monitor the daily food intake. Therefore, our study was unable to account for changes in food intake that might accompany voluntary wheel running, which may significantly contribute to variation in gut microbiome.

We recognize the significance of sex as a biological variant and acknowledge that only including male rats in this study is a limitation. Existing literature and our own data indicate that while the running distance of rats largely increases over time, female rats run significantly more than males under the same conditions and exhibit estrus cycle-dependent variation in voluntary wheel running (VWR) activity (Jones et al., 1990; Lightfoot, 2008; Konhilas et al., 2015; Rosenfeld, 2017; Mathis et al., 2024). This difference has been linked to the female sex hormone estrogen and its receptors (Ogawa et al., 2003; Gorzek et al., 2007; Mathis et al., 2024). However, in practical conditions, the female estrus cycle is not synchronized within a cohort, which serves as a potential confounding factor. We have recently adopted a method (Borjeson et al., 2014) to synchronize their estrus cycle in female rats. Given that female rats outperform males in VWR, we anticipate identifying even more pronounced and robust microbiome changes associated with wheel running. Additionally, it is possible we may identify female-specific changes in the gut microbiome that are linked with estrogen.

## 5 Conclusion

This study identifies that changes to the microbiome occur within the same timeframe as exercise adaptation. This suggests that the early exercise period is the most crucial timeframe for GMB remodeling, and that adaptation during this period is the best predictor of terminal behavior. Future works should continue to characterize the biologic and microbiome cascade which occurs in this early period to identify pathways that can be up regulated to enhance performance. *R. bromii* was identified as a candidate for augmenting the adaptive period. Consistent with previous research, *Peptostreptococcaceae* was identified as a detrimental feature of the GMB which both reduced alpha diversity and was associated with decreased running behavior. It is not clear whether the presence of *Peptostreptococcaceae* itself contributed to this observation, or whether it was decreased alpha diversity was the greater contributor, though our modeling suggests *Peptostreptococcaceae* may be the greater contributor. These results suggest that microbiome adaptation during voluntary exercise behavior is an important factor in adaptation.

## Data availability statement

The datasets presented in this study can be found in online repositories. The names of the repository/repositories and accession number(s) can be found below: https://www.ncbi.nlm.nih.gov/, SUB14151585; https://www.ncbi.nlm.nih.gov/, PRJNA1078395.

## Ethics statement

The animal study was approved by Des Moines University, Des Moines IA. The study was conducted in accordance with the local legislation and institutional requirements.

## Author contributions

MR: Conceptualization, Data curation, Formal analysis, Investigation, Software, Visualization, Writing – original draft, Writing – review & editing. AnK: Data curation, Formal analysis, Visualization, Writing – original draft, Writing – review & editing. AvK: Writing – original draft, Writing – review & editing. CL: Investigation, Writing – review & editing. LW: Investigation, Writing – review & editing. VM: Investigation, Writing – review & editing. TL: Formal analysis, Writing – review & editing. LY: Conceptualization, Formal analysis, Funding acquisition, Investigation, Methodology, Project administration, Supervision, Visualization, Writing – original draft, Writing – review & editing.
